# Morphological, Physiological and Molecular Markers for Salt-Stressed Plants

**DOI:** 10.3390/plants10020243

**Published:** 2021-01-27

**Authors:** Aigerim Soltabayeva, Assel Ongaltay, John Okoth Omondi, Sudhakar Srivastava

**Affiliations:** 1 Biology Department, School of Science and Humanities, Nazarbayev University, Nur Sultan Z05H0P9, Kazakhstan; assel.ongaltay@nu.edu.kz; 2 International Institute of Tropical Agriculture, PO Box 30258 Lilongwe 3, Malawi; okoth05@gmail.com or; 3 Beijing Advanced Innovative Center For Tree Breeding by Molecular Design, Beijing Forestry University, No. 35, Qinghua East Road, Beijing 100083, China; srivastavasudhakar@gmail.com

**Keywords:** salinity stress, stress tolerance, morphological markers, physiological markers, chlorophyll, antioxidant, molecular markers

## Abstract

Plant growth and development is adversely affected by different kind of stresses. One of the major abiotic stresses, salinity, causes complex changes in plants by influencing the interactions of genes. The modulated genetic regulation perturbs metabolic balance, which may alter plant’s physiology and eventually causing yield losses. To improve agricultural output, researchers have concentrated on identification, characterization and selection of salt tolerant varieties and genotypes, although, most of these varieties are less adopted for commercial production. Nowadays, phenotyping plants through Machine learning (deep learning) approaches that analyze the images of plant leaves to predict biotic and abiotic damage on plant leaves have increased. Here, we review salinity stress related markers on molecular, physiological and morphological levels for crops such as maize, rice, ryegrass, tomato, salicornia, wheat and model plant, *Arabidopsis*. The combined analysis of data from stress markers on different levels together with image data are important for understanding the impact of salt stress on plants.

## 1. Introduction

Growth and development of plants are affected by various stresses. Salinity is one of the major abiotic stress which adversely affects the overall growth and yield of crops [1,2,3]. It is estimated that >1 billion ha of the world land is salinized [4] and continued salinization of the ever-decreasing agricultural land further exacerbates food insecurity as human population surges. Some of the major world crops such as maize, wheat, rice, tomato and sunflower are reviewed here where, salinity resulted in the reduction of the yield [2,5,6,7,8]. The compromised performance causing poor yield could be due to the reduction in photosynthesis efficiency, chlorophyll, total protein, biomass, stomata closure and increasing the oxidative stress [9].

To improve productivity in salt-affected soils, selection and adoption of plant varieties with high salt tolerance has always been a preferred choice [10,11]. This selection is based on morphological, physiological and molecular markers. Among morphological markers, root or shoot morphology, visible early senescence, biomass of grains is some of the important parameters that are considered. Physiological and biochemical markers examine chlorophyll content, accumulation of proline, sucrose, stress protectants, membrane stability and hormones content [9,12]. These physiological markers, especially hormonal, polyamine and proline changes in plants are important to increase salt tolerance of plants. For example, such can be boosted by exogenous treatments with hormones, glycine betaine, proline, polyamines, paclobutrazol, nanoparticles [13]. The molecular markers include salt stress tolerant genes, transcription factors, metabolic pathway related genes [9,12]. These molecular markers have led to significant progress in genetic engineering of plants with salt tolerance [9]. Altogether, all stress markers in plants help in identification of specific genes involved in salt tolerance [9].

The advent of information technology (IT), especially different smart data analysis techniques which are being applied in other fields, are also directed towards the improvement of agriculture. These used techniques are known as machine learning—a subset of artificial intelligence. Machine learning algorithms build a model based on data samples, called “training data,” developed in order to make predictions or decisions without being explicitly programmed to do so [14]. Deep learning is a machine learning method based on artificial neural networks with multiple hidden layers and representation learning [15]. Deep learning architectures have been applied to various fields including agriculture, bioinformatics, drug design, medical image analysis, among others, where they predict results with a given data set and have produced comparable results and, in some cases, surpassing performance of human experts [15,16,17]. Deep learning algorithms such as Convolutional Neural Networks (CNN), Recurrent Neural Networks (RNN), Long Short-Term Memory (LSTM) are frequently applied for prediction and/or detection, quantification of biotic stresses in plant leaves [17,18]. They are used for screening stress tolerant or sensitive genotypes by using the morphological characteristics of plants through recorded images [19]. Few of these studies are related to abiotic stress, for example drought [20,21,22] and salinity [19]. This evaluation and prediction of stress impact on plants by deep learning approaches give additional agricultural solutions to prevent the risk of yield losses through abiotic or biotic stresses. Thus, they have a big potential in developing smart greenhouse or field production. However, this approach primarily records morphological markers as it only analyzes the image-type data. This limits the applicability of IT in rigorous salinity studies and necessitates the knowledge of additional markers that can explain the mechanism at molecular level. Different salinity stress markers in plants at physiological, metabolic, protein and molecular levels result in near-conclusive understanding of the salinity effects, challenges and solutions. Therefore, this review focuses on elaborating advances in salinity stress markers that lead to recognition of salt stress in plants at morphological, physiological and molecular level, as well as on early detection methods and salinity stress symptoms that are commonly used in the plant biology research community.

## 2. Detection of Salinity Stress

### 2.1. Morphological Markers

**Figure 1:**
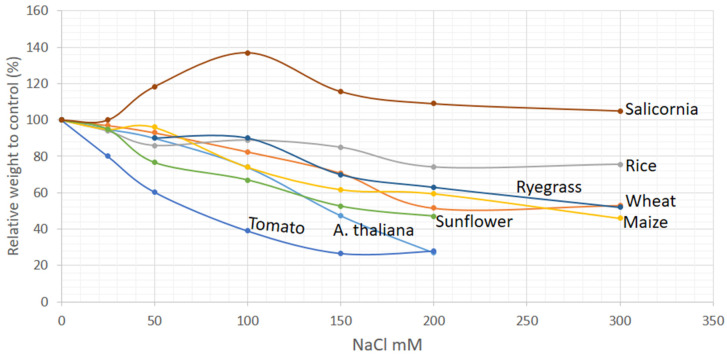
Effect of salinity level on relative biomass changes in crop plants. The relative dry weight compared to control was used as a growth parameter. The irrigation of soil by saline water (NaCl) from 0–300 mM was used salt application. Sources of the data were: *A. thaliana*: [25,32,41,42], wheat: (*Triticum aestivum*) [30,36,43,44], rice (*Oryza sativa*): [31,33], maize (*Zea mays*): [24,34,45,46,47], tomato: (*Solanum lycopersicum*): [35,48,49], sunflower: (*T Helianthus annutis*): [50,51,52], ryegrass (*Lolium perenne*): [53], salicornia: [37,38,39].

Salt concentrations in soils vary from one place to another and that affects the growth of plants depending on the concentration [12]. Generally, NaCl salt is commonly used for salt stress studies [12] and in this review we focused on NaCl induced stress signs in plants. The stress effects on morphology are manifested by different means such as dry or fresh total biomass, plant height and through other morphological markers [23,24,25]. Generally, an increase of salt content in the growing environment increases the impact of salt stress on plant growth (Figure 1). This response varies from plant to plant [12]. In *Arabidopsis*, wheat, maize, rice and rye grass, the decrease of total plant biomass was observed at 100–150 mM NaCl levels, while in tomato and sunflower, the weight decreased on 50 mM NaCl application (Figure 1). Studies on salinity effect on trees are scarce, even though they too are affected by salinity, in citrus and acacia, a 100 mM NaCl caused biomass changes (decrease of 13% and 15%, respectively) at early stages of growth [26,27,28], however, palm was less susceptible as it experienced growth changes (biomass did not decrease) at severe salt stress levels [29]. Despite the differences observed in biomasses in response to salinity levels, the growth of all these plants were significantly repressed by 200–300 mM salt (NaCl) treatment [30,31,32,33,34,35,36]. Conversely, these salt concentrations did not affect the growth of halophyte plants such as salicornia (Figure 1). However, above 400 mM NaCl, the yield of a salicornia is also decreased [37,38,39,40].

**Figure 2:**
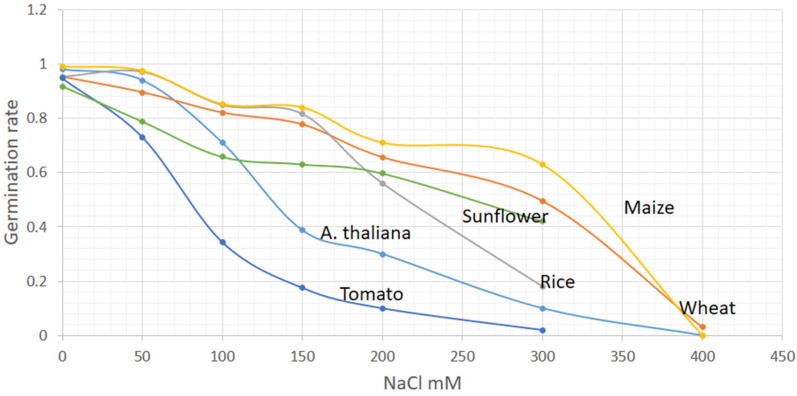
Changes in germination rate of crop plants at different levels of salt stress. Germination rate after 7 days plants grown under different level of NaCl concentration. Data presented for *A. thaliana* [54,55,56,57], wheat (*Triticum aestivum*) [24,47,58,59], rice (*Oryza sativa*) [31,60], maize (*Zea mays*) [46,61,62], tomato (*Solanum lycopersicum*) [63,64,65], sun flower (*T Helianthus annutis*) [50,66], salicornia [39,67].

Salinity stress also affects germination rate of seeds and thus germination is also an informative marker for salinity stress. Since germination is among the foremost morphological processes, it is a useful indicator of stress as compared to biomass as stress can be known as early as 2–14 days depending on a plant species. The germination rate of *Arabidopsis*, sunflower and tomato declined by 71%, 62% and 35%, respectively at 100 mM NaCl (Figure 2). The germination percentage of wheat, maize and rice declined significantly at higher salinity level (200 mM) compared to biomass changes (Figure 1 and Figure 2). At 400 mM NaCl, salinity stress is critical and most crops do not germinate, although below this concentration, some plants such as wheat can have 3% germination rate with a retarded growth (Figure 1 and Figure 2). Thus, the growth markers in plants are sensitive to salt stress at 100–400 mM NaCl.

In addition to total plant biomass changes, the weight of shoot, root and leaves are frequently used for evaluation of salt stress [12]. The length of roots and shoots, root architecture and the number of secondary branches on them, diameter of shoot, the tiller numbers, leaves number are used as growth parameters in salinity [23,26,34,35,42,44,45,47,68,69]. Generation rate of young leaves is also used for analysis of salinity impact [12,32]. Besides the growth parameters, the flowering time may show the effect of salt stress on the reproductive stage of plants. In *Arabidopsis*, wheat, barley, maize and rice the flowering is delayed under salinity stress [70,71,72,73,74,75,76,77].

### 2.2. Physiological Markers

Measurement of Na^+^ or Cl^−^ ions concentration in the leaves and roots reflect salinity stress in plants (Table 1). Measurement of potassium ion (K^+^) content and/or ratio of K^+^/Na^+^ are also frequently used (Table 1). In addition, ratios of other ions such as Na^+^/Ca^2+^, Ca^2+^/Mg^2+^ and Cl^−^/NO_3_^−^ are usually evaluated as they influence nutrient uptake [12]. Increase of other salts in the soil in addition to available 50–100 mM NaCl alters the intracellular concentration of salt (Na^+^ and Cl^−^) in *Arabidopsis*, wheat, rice and maize [23,24,32,68,78].

Salinity stress decreases photosynthesis process [12]. This is evident by monitoring stomatal conductance, chlorophyll fluorescence and chlorophyll contents [12,79]. The decrease in chlorophyll content was observed under 50 mM–250 mM salt application in major crop plants (Table 1). The osmotic parameters of plant changes rapidly in response to salt stress [12]. This is usually expressed by evaluating the changes in turgor pressure, osmotic pressure, RWC and water potential [33,37,39,78]. For example, water potential in *Arabidopsis* decreases upon treatment with 100 mM NaCl (Table 1). Another way of evaluating osmotic changes occurring during salinity stress is the measurement of osmolytes such as sucrose, proline, glycine-betaine. These osmolytes are stress protectors and their accumulation in plants experiencing salinity stress is an adaptive mechanism [12]. Sucrose and proline normally increase in salinity levels of 75–200 mM, however, this varies among plants; for example, in *Arabidopsis*, wheat and rice it is at 75–200 mM NaCl application, while in maize it is at 100 mM [32,33,34,80,81,82].

### 2.3. Oxidative Stress Markers

Salinity stress causes imbalance of reactive oxygen species [112]. This imbalance is mainly as a consequence of disruption of electron transport chains (ETC) during photoinhibition and/or decrease in water potential [113]. The ROS is dramatically increased upon salinity stress and the first ROS reaction is termed as “oxidative burst” [114]. The higher level of ROS becomes toxic for cells resulting in cellular damages and may lead to its death, if increase is unchecked. Moreover, ROS also acts as signaling molecule that may lead to the changes in transcript, proteins and metabolites in order to activate some of the adaptive mechanisms [112]. Since different plant species have different sensitivity towards salinity, the imbalance in ROS level is also detected at different salt concentration, for example, 50–100 mM salinity level in *Arabidopsis,* tomato, wheat, rice, maize plants, 120 mM NaCl in sunflower, 255–300 mM in ryegrass, 400 mM–600 mM in salicornia [87,115,116,117,118,119,120,121]. In addition to the concentration, duration of salinity is also a crucial factor in determining the alteration in cellular ROS, for example, *Arabidopsis*, tomato and rice show ROS imbalance after several hours of salt treatment [87,122,123,124,125,126,127,128] while in wheat, maize, sunflower, ryegrass, salicornia the ROS level significantly increases after several days [107,121,129,130,131,132]. Additionally, higher changes in ROS under salinity is observed in older leaves as compared to younger leaves of rice and maize [133,134].

It is known that ROS level is regulated by enzyme activity of ROS producers and ROS scavengers [112]. Parallel to these ROS changes in plants during the salinity stress, there is increase of peroxidase (POD), catalase (CAT), superoxide dismutase (SOD), glucose-6-phosphate dehydrogenase (G6PDH), ascorbate peroxidase (APX), glutathione S-transferases (GST), glutathione peroxidases (GPX), glutathione reductase (GR), dehydroascorbate reductase (DHAR), monodehydroascorbate reductase (MDHAR), polyphenol oxidase (PPO), phospholipid hydroperoxide glutathione peroxidase (PHGPX) in mature leaves of tomato, *Arabidopsis*, wheat, rice, maize, salicornia (Table 2). These ROS related enzyme activity changes are determined under similar concentration and time point of salt stress. Additionally, it was shown that NADPH oxidases are important in plant response during salinity stress [135]. Apart from enzymatic antioxidants, there is also an increase in non-enzymatic antioxidants such as AsA (Ascorbate), GSH (glutathione) and tocopherols as observed in *Arabidopsis*, wheat, sunflower; ryegrass, salicornia [87,89,107,121,132,136,137,138,139].

Another marker of salt stress is cell membrane injury and this can be determined by electrolyte leakage and/or higher water loss (a decrease of RWC) [140,141] (see Table 1). The cell membrane damage is generally due to the enhancement in ROS production during the salt stress [9]. Also, lipid peroxidation marker such as Malondialdehyde (MDA) content increase under salinity stress in *Arabidopsis*, wheat, rice, tomato, maize, ryegrass, salicornia [37,87,116,117,129,132,142,143,144,145,146,147,148]. The increase in MDA in plants experiencing salinity is detectable after the ROS molecule and enzyme activity changes. In rice and maize, it is more in older leaves than younger ones [93,133,134]. Additionally, the instability of the membranes may be visualized by thermography and hyperspectral reflectance techniques measurements [11,19]. These techniques are based on the abilities of plants to reflect and absorb light at different wavelengths (Raman, near-infrared, fluorescence etc.) depending on biophysical characteristics of the plant cell, such as disruption of electron transport chains (ETC), which changes under different stresses [149]. These changes in oxidative stress markers in plants such as ROS molecules, ROS related enzyme activity, MDA and membrane stability (leakage or RWC) are triggered not only by salt stress but other stresses as well. However, these changes are mainly detectable in early stress response of plants and in combination with morphological salt stress markers, they could be used for early predictions of salt stress in plants.

### 2.4. Molecular Stress Markers

Transcriptomic and proteomic studies reveal that stress triggers the transcript and proteome changes in plants [9]. From transcript analysis for plants under salinity stress, some of the genes change their expression under various salt concentrations. These groups of genes could be used as molecular markers for prediction and confirmation of salt stress in plants. In Table 2 and Figure S1 some of the genes that frequently show difference in expression during the salinity stress (NaCl applications) are presented.

Most of the genes used for evaluating salinity stress impact in plants are involved in cell protection, membrane transporters, osmoprotectants, ROS regulation enzymes and signal transductions (e.g., transcription factors and protein kinases) (See Table 2 and Figure S1). Also, some of these genes are associated with senescence such as *NAC, NAP, WRKY 53, WRKY 25, SAG12, GDH* (Table 2 and Figure S1). Measurements of genes expression from different functional groups such as ion homeostasis, biosynthesis of osmoprotectants, polyamines, synthesis of ROS scavengers and antioxidant enzymes and senescence process related genes in salt stressed-plants allow better confidence in prediction of stress impact in plants and could be used to predict the mechanism by which plants respond to salt stress. Thus, based on transcriptional changes of genes, one may predict the changes at physiological and morphological level. Additionally, the high-throughput phenotyping is identifying the genetic components of salinity tolerance [150], which could also be used as molecular markers.

The molecular markers for evaluation of stresses are rarely used in research for trees and other non-model plants compared with the morphological and physiological measurements, which are less expensive and easy to conduct. However, it might be possible to generate molecular markers for less/rarely studied plants based on homologues of stress related genes that were studied well in other model or important crop plants (Table 2).

## 3. Evaluation of Salinity Stress in Plants by Different Stress Markers

Depending on the concentration and duration, generally, salinity affects all the plants, some of which, like *Arabidopsis* and tomato are more sensitive, whereas others such as wheat, rice, rye grass and so forth are less sensitive (See Figure 1 and Table 1). Nevertheless, the changes at molecular, physiological, morphological level under salinity stress have similar trends (either increase or decrease) for the discussed crop plants. The measurements of Na^+^ and K^+^ ions content in plants give strong proof for salinity stress. Other stress signs may also provide the information related to salinity strength and time of exposure. For example, the morphological stress markers such as relative weight changes and germination may predict the moderate and toxic level of salinity, respectively (Figure 1 and Figure 2). Monitoring the morphological changes coupled with Machine learning approaches could prevent salt stress in plants in smart greenhouse. In addition, evaluating salt sensitive (Tomato or *Arabidopsis*) and tolerant (salicornia) plants side-by-side in a smart greenhouse could reliably predict the ability of the examined plant to tolerate the extent of salt stress. For example, if the plant being studied suffers similar to tomato or *Arabidopsis* while salicornia growth remains unaffected, the salinity level in the soil is likely to in the range of 100–300 mM NaCl (Figure 1 and Table 1).

The physiological traits such as chlorophyll content, RWC, electrolyte leakage, stomatal conductance, water potential, proline, glycine betaine change on application of 100 mM NaCl after several days (Table 1). The molecular markers manifest the 100 mM salt stress level prior to physiological changes. These can be detected in minutes or longer time (Table 1 and Table 2). The ROS changes and ROS related enzyme changes are also early recognizable markers for stress [87,122,123,124,125,126,127,128]. Generally, the ROS level changes have wave shape changes during the time of stress exposure, this fluctuation of ROS occurs in stressed and also in normal conditions [179,180]. Thus, to define the fluctuation in ROS level under moderate stress, it is better to measure ROS level in time-series rather than image-type measurements, which is a snapshot measurement only for one time point [181]. Spectrometric or staining methods are commonly used for ROS detection under salinity stress [182] but also the imaging system are rapidly developing for monitoring redox state of the plant cell [183,184,185] allowing measurement of ROS changes in vivo [186].

**Figure 3:**
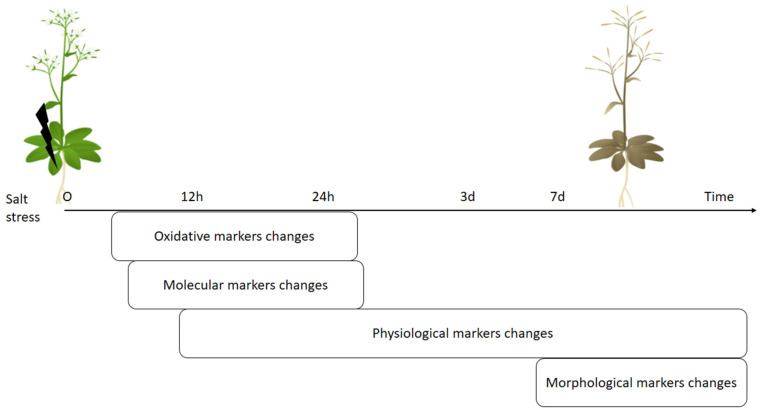
Scheme of sequence of changes at different levels in plants triggered by salt stress.

Basically, these molecular, physiological and morphological changes in plants follow the order, where the first changes by stress will be visualized by molecular, followed by biochemical then physiological and at last by morphological markers (Figure 3, Table 1 and Table 2). Additionally, the ROS molecule plays an important role in signaling for stress and thus, these oxidative stress markers changes are detectable at similar time with molecular markers changes after exposure by salt application (see time points in References [87,122,123,124,125,126,127,128] and Table 2). Therefore, each stress marker has order in terms of time observation after stress, where the oxidative and molecular stress markers are early sensors for stress compared with other markers but they will not specify stress type.

Additionally, if the measurements will focus on specific locations in plant organs like young and/or older leaves then the recognition of stress in plants will be detectable in early stages. For example, the old leaves are accumulating salt earlier than young leaves in crop plants (Table 3). Moreover, the visible symptoms of senescence such as decrease in total chlorophyll and/or chlorophyll a, b content and decrease of photosynthesis efficiency was also higher in old leaves in comparison to young leaves of crop plants upon NaCl application (Table 3). Chlorophyll content measurements, fluorescence were also greater decreased in old leaves under salt stress [93,187] and this measurement does not require the detachment of the leaves (Table 3). Additionally, the degradation of total soluble proteins was higher in old leaves as compared to young leaves under salinity stress (Table 3). Also, it was shown that the ROS levels and catalase activity were higher in old leaves than young leaves after salt stress applications (Table 3). Other signs of senescence such as ethylene gas production was observed after salt treatments [90]. Also, the remobilization of nitrogen and the formation of the autophagosome under salinity stress may also be used as markers for the salinity stress in plants [188]. Generally, the senescence symptoms in leaves could be detected in earlier stages by using molecular markers (Table 2 and Figure 3). Thus, some morphological, physiological and molecular changes under salt stress are highly detectable in older (mature) leaves compared to other organs or whole plant and measurements of these stress markers on a specific area of plants would increase efficiency of deep learning approaches on phenotyping the salt stressed plants.

## 4. Prediction and Identification of Stressed Plants Using Deep Learning Approaches

Machine learning techniques are developing rapidly for agricultural needs such as for plant recognition, plant or fruits counting, classification of crop types, phenotyping of various plant species, classification of mutants, leaf counting, identification of vein patterns and leaf characteristics detection of plant diseases, weed control, as well as the prediction of biotic stresses in plant leaves [17]. Basically, these approaches analyze big data of images, from monitoring various morphological changes in plants to identifying and/or classifying and/or phenotyping plants [17,196]. There are so many different machine learning approaches out there but some are frequently applied in plants, such as Artificial Neural Networks (ANN), Logistic Regression, Random Forest, Support Vector Machines (SVM), K-Nearest Neighbors (KNN) and Deep Learning are commonly used in prediction and/or classification and/or detection of stress in plants [17,197,198]. Among these different machine learning approaches, the deep learning models such as Convolutional Neural Networks (CNN), Recurrent Neural Networks (RNN), Long Short-Term Memory (LSTM) are recently on the increase for use in imaging analysis. This is because the CNN has shown great accuracy in finding specific patterns in image data, so it is mainly used for identification and classification of different damages in plant leaves, especially for searching the damages caused by biotic and abiotic stimuli [17]. The other models, RNN and LSTM, are also valuable in the analysis of time series image data [17], which is important for prediction of damages in plant leaves. It has also been pointed out that various combinations of deep learning approaches can be used for classification and prediction of plant characteristics [17,199] and these combinations of different models can be used in the future for accurate diagnosis of signs of stress in plants for smart greenhouse procedures.

Generally, all these high-throughput phenotyping technologies are based on the analysis of different type of images such as RGB imaging, near-infrared imaging, infrared thermal imaging and fluorescence imaging [200,201,202]. These prediction of plant diseases and pest attacks or environmental impact on plants by machine learning approaches are mostly focused on identification of visual symptoms of biotic damage [17], which are discussed as morphological stress markers. As mentioned above, these morphological signs appear in plants later than physiological, oxidative and molecular stress markers (Figure 3). Currently, deep learning approaches are beginning to combine morphological stress markers data (visible signs in leaves) with physiological stress markers such as transpiration rate, biomass, water content, biochemical components (sodium concentration), photosynthetic efficiency, caratenoides [19,203,204,205]. However, to the best of our knowledge, there is still no extensive research on deep learning approaches for predicting abiotic or biotic stress in plants that use a combination of oxidative, molecular and morphological markers. In addition, using deep learning analysis for attached or detached leaves and whether these leaves are mature or young for better prediction has not been done yet. As previously mentioned, physiological and morphological stress marker analysis of older leaves (lower leaves) shows greater changes under salinity stress rather than young leaves (Table 2) but it has not yet been shown how this could affect prediction or identification damage using deep learning approaches. In addition, deep learning approaches for predicting salinity stress in plants have only been applied for a single plant species (Barley or Spinach or Okra) [19,20,21,22]. It would be interesting if machine learning approaches were applied in different plant species, specifically to the salt sensitive (Tomato or *Arabidopsis*) and tolerant (salicornia) plants, for their identification and prediction of salinity stress in plant (Figure 1 and Table 1). Additionally, it successfully generated different transgenic plants with different gene modifications, for example, it generated fused constructions of the gene promoter region with GFP proteins [206]. These transgenic plants (in Table 2) with GFP protein, could be useful for evaluation of molecular stress markers and prediction of salinity stress in plants. Thus, we believe that the following suggestions have potential for application through deep learning approaches for stress prediction in plants: (a) analysis of images data with other stress markers such as oxidative and/or molecular markers, (b) emphasis on analysis of mature leaves versus young leaves, (c) use of control plant data such as stress-sensitive or stress-tolerant plants or the transgenic plant promoter fused with GFP and other fluorescent markers.

## 5. Conclusions

Continuous expansion of soil salinization area is inversely proportional to crop yield and this poses a challenge to global food security. To improve plant adaptability and performance to such condition, different areas such as plant stress physiology, molecular biology, genetic engineering, biotechnology are being exploited. Machine learning approaches are promising in developing smart greenhouse, by phenotyping plants and controlling the environmental growth parameters. Developing such controlled growth room conditions require not only equipped imaging technologies but also important physiological, oxidative and molecular data. Morphological markers in plants such as root or shoot growth, germination, flowering time indicate obvious signs of stress but also the appearance of senescence symptoms under salinity stress is an important sign—which appear early in older leaves after salt application. From physiological parameters, the chlorophyll content, RWC, electrolyte leakage, stomatal conductance, water potential, proline, glycine betaine changes in plants are commonly detected under NaCl stress. These physiological changes manifest themselves before the molecular markers. ROS and ROS-related-enzyme changes are also early recognizable markers for stress. Molecular and oxidative stress maybe useful in early detection of salinity stress impact. Still, each stress marker, either morphological, physiological, oxidative or molecular changes in plants, have their own limitation. An integrated approach and usage of different sensors for specific areas of the plant such as old leaves would increase the sensitivity in detection of salinity stress in plants. However, this integration of morphological, physiological, molecular and deep learning parameters requires concerted studies.

## Figures and Tables

**Table 1:** Range of changes of the physiological parameters depend on time and concentration of NaCl application.

Physiological Parameters	Salt Sensitive Plants		Less Salt Sensitive Plants					Halophyte
	Tomato	*A.Thaliana*	Rice	Maize	Wheat	Sunflower	Ryegrass	Salicornia
Na content increase	50–100 mM; 1 d	100 mM; 4 h–1 d	100 mM; 6 h–7 d	100 mM; 21 d	100 mM; 3 d	100–150 mM; 1 d	100–250 mM; 4 d	100–200 mM; 10–42 d
K content decrease	50–100 mM; 1 d	100 mM; 4 h	100 mM; 7 d	-	75–100 mM; 7–42 d	100 mM; 1 d	100–250 mM; 4 d	100–200 mM; 10–42 d
RWC * decrease	100 mM; 2 h	100–200 mM; 1–15 d	100 mM; 4–12 h	50 mM; 7 d	75–100 mM; 3–42 d	50–100 mM; 2–54 d	200–250 mM; 12–14 d	100–200 mM; 10–42 d
Chlorophyll decrease	75–100 mM; 3 d	100–200 mM; 4–18 d	30–150 mM; 6 h–3 d	50 mM; 21 d	50–100 mM; 3–42 d	100 mM; 40–60 d	250 mM; 14 d	85–250 mM; 14–30 d
Electrolyte leakage increase	50–100 mM; 1 d	100 mM; 6 h–15 d	100 mM; 6 h	100 mM; 8 d	100 mM; 30 d	100 mM; 2 d	100–250 mM; 12–14 d	-
Stomatal conductance decrease	100 mM; 7–12 d	100 mM; 15 d	100 mM; 30 d	60 mM; 12 h	100 mM; 7–21 d	50–100 mM; 5–35 d	-	-
Water potential	100 mM; 2 h	100 mM; 4 d	100 mM; 48 h	60 mM; 20 d	100 mM; 5–70 d	25–100 mM; 1–54 d	-	150–400 mM; 24–42 d
Proline increase	-	100 mM; 14–15 d	-	50 mM; 1 d	50–100 mM; 7–42 d	100–150 mM; 7–15 d	-	85–600 mM; 30–45 d
Glycine betaine increase	-	-	-	50 mM; 21 d	-	100 mM; 7 d	-	200–500 mM; 15 d
**Reference**	[83,84,85,86]	[87,88,89,90,91]	[92,93]	[94,95,96,97]	[98,99,100,101,102]	[103,104,105,106]	[107,108]	[109,110,111]

* RWC = relative water content; h = hours; d = days.

**Table 2:** List of genes, which change their expression under salinity treatments depending on the salt concentration and time of application.

Groups/Class		Genes	Inductions of Genes under Salinity Conditions		Plant Species	Reference
			NaCl Concentrations	Time		
Stress sensors	Ca sensor kinase	*CIPK11* *CIPK21*	150 mM	24 h	*A. thaliana*	[151]
		*CBL2*	250 mM	24 h	*T. aestivum*	[152]
	Protein kinase	*SOS2* *SOS3*	100–200 mM	3–6 h	*A. thaliana*	[153,154,155]
Ion balance regulators	Na transporters	*NHX1*	100 mM–250 mM	3 h–21 d	*Salicornia*, *T. aestivum*, *O. sativa*	[38,152,156,157]
	Chloride anion channel	*CLC*	150–200 mM	3–9 d	*O. sativa*, *A. thaliana*	[157,158]
	Cation channel	*ERD4* *GLRX*	200 mM–300 mM	24 h–5 d	*A. thaliana*; *T. aestivum*	[159,160]
	Transferases	*GST*	150 mM–255 mM	6–48 h	*T. aestivum*, *A. thaliana*, *R*	[152,161,162,163]
ROS regulators	ROS scavengers	*APX*	50–255 mM	48 h–15 d	*T. aestivum*, *O. sativa*, *Z. mays*, *S.lycopersicum*, *Ryegrass*	[30,45,161,164,165]
		*CAT*	25–255 mM	48 h–7 d	*T. aestivum*, *Z. mays*, *S. lycopersicum*, *O. sativa*, *Ryegrass*	[30,45,157,161,164,165]
		*SOD*	50–250 mM	4–75 d	*T. aestivum*, *S. lycopersicum*, *Ryegrass*	[30,164,165]
	Redox enzymes	*GR*	100–150 mM	24 h–7 d	*A. thaliana*, *T. aestivum*	[30,162]
		*LOX*	300 mM	5 d	*T. aestivum*	[160]
		*PAO*	50–250 mM	3 h–5 d	*T. aestivum*, *A. thaliana*	[160,166]
Cell protectors	Proline synthesis	*P5CR*	100 mM–250 mM	2 h–6 d	*O. sativa*, *T. aestivum*, *S. lycopersicum*	[167,168,169]
		*P5CS*	100 mM–255 mM	2 h–6 d	*A. thaliana*, *O. sativa*, *S. lycopersicum*, *Ryegrass*	[161,167,168,170,171]
	Polyamine biosynthesis	*ADC*	100–250 mM	5–7 d	*T. aestivum, O. sativa*	[160,172]
Senescence regulators	Transcriptional factors	*NAC*	150 mM–200 mM	12 h–24 h	*A. thaliana*, *Salicornia*	[162,173]
		*NAP*	150–200 mM	5 h–6 d	*A. thaliana*, *O. sativa*	[174,175]
		*ANAC092*	150 mM	6–24 h	*A. thaliana*	[151,176]
		*WRKY53*	150 mM	4 d	*A. thaliana*	[177]
		*WRKY25*	150 mM	6 h	*A. thaliana*	[162]
	Protease	*SAG12*	150 mM	4 d	*A. thaliana*	[176,177]
	Nitrogen remobilization	*GDH2*	100–150 mM	48 h–6 d	*O. sativa*, *A. thaliana*	[163,178]

**Table 3:** Differences in old and young leaves of crop plants triggered by salinity stress.

Parameters	Plants	References
Higher accumulation of Na^+^ in old leaves than young leaves	Wheat, Barley, Rice, Maize, Sunflower	[93,133,134,187,189,190,191,192,193]
Higher accumulation of Cl^−^ in old leaves than young leaves	Wheat and Barley, Sunflower	[187,189,191]
Higher photochemical efficiency decrease in old leaves than young leaves	Wheat and Barley, Rice	[93,187]
Higher decrease of total chlorophyll content in old leaves than young leaves	Wheat, Barley, Sunflower	[93,192,194]
Higher soluble protein decrease in old leavesthan young leaves	Rice, Wheat	[93,195]
Higher increase in MDA content in old leaves than young leaves than young leaves	Rice, Maize	[93,133,134]
Higher electrolyte leakage in older leaves than young leaves	Rice, Maize	[93,134]
Higher ROS reduction and H_2_O_2_ generation in older leaves than young leaves	Rice, Maize	[133,134]
Higher increase in catalase activity in older leaves compare to young	Rice, Maize	[133,134]

## Data Availability

Data is contained within the article or supplementary material.

## Supplementary Materials

The following are available online at https://www.mdpi.com/2223-7747/10/2/243/s1, Figure S1: Change in expression of genes in *Arabidopsis*, wheat, rice and maize triggered by salt stresses.
