# Supplementary material for: Morphological, Physiological and Molecular Markers for Salt-Stressed Plants
Source: Plants (Basel). 2021 Jan 27;10(2):243. doi: 10.3390/plants10020243 (PMC7912532; doi:10.3390/plants10020243)
Supplement: Supplementary file 1 [file plants-10-00243-s001.pdf]

Supplementary Materials

Morphological, physiological and molecular markers for salt-stressed plants

Aigerim Soltabayeva [1,*], Assel Ongaltay [1], John Okoth Omondi [2], and Sudhakar. Srivastava [3]

[1] School of Science and Humanities, Biology Department, Nazarbayev University, Astana, Z05H0P9, Kazakhstan; aigerim.soltabayeva@nu.edu.kz and assel.ongaltay@nu.edu.kz

[2] International Institute of Tropical Agriculture, Lilongwe, Malawi; okoth05@gmail.com; jo.omondi@cgiar.org

[3] Beijing Forestry University, People Republic of China; srivastavasudhakar@gmail.com

* Correspondence: aigerim.soltabayeva@nu.edu.kz

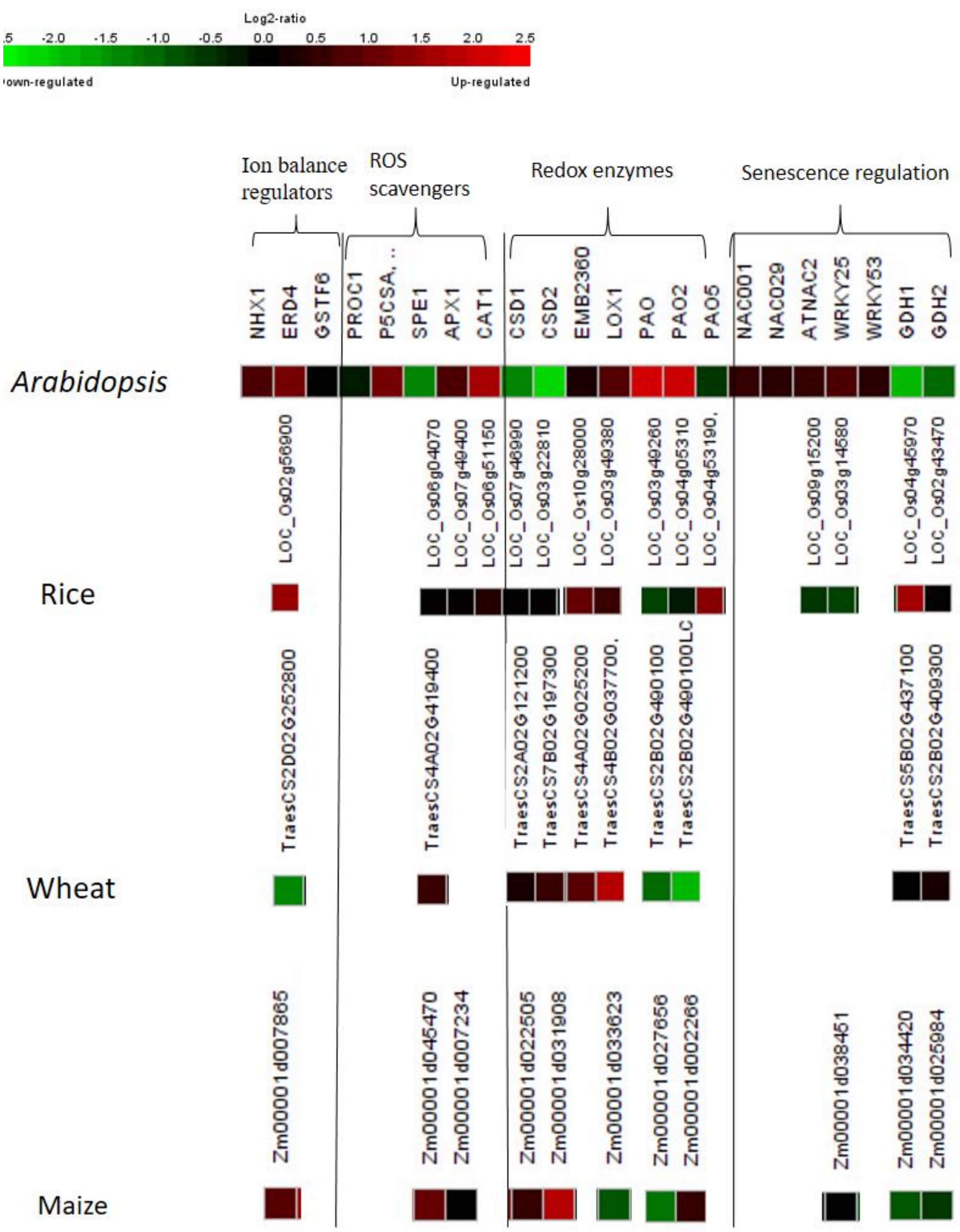

Figure S1. Change in expression of genes in *Arabidopsis*, wheat, rice and maize triggered by salt stress. Inspection of the transcripts responses was performed in published microarray collections (GENEVESTIGATOR; https://www.genevestigator.ethz.ch). The analysis is presented in Log2 view (fold change in expression relative to control treatment), and the results are presented as a heat map. The transcript analysis done with genes involved in ion balance regulation, ROS scavengers, Redox enzymes and senescence regulatory genes (list of genes was collected from Table 4) in shoots part of *Arabidopsis* and rice plants after 3 hours and wheat (Chinese Spring) and maize after 24h of NaCl applications. The functional orthologs of Arabidopsis genes were retrieved from maize, wheat and rice using the GENEVESTIGATOR Ortholog Search Tool.
